# Chlorogenic Acid as a Positive Regulator in LPS-PG-Induced Inflammation via TLR4/MyD88-Mediated NF-*κ*B and PI3K/MAPK Signaling Cascades in Human Gingival Fibroblasts

**DOI:** 10.1155/2022/2127642

**Published:** 2022-04-09

**Authors:** Chung Mu Park, Hyun-Seo Yoon

**Affiliations:** ^1^Department of Clinical Laboratory Science, Dong-Eui University, Republic of Korea; ^2^The Research Institute for Functional Health Materials, Dong-Eui University, Republic of Korea; ^3^Department of Dental Hygiene, Dong-Eui University, Republic of Korea

## Abstract

Gingival inflammation is one of the main causes that can be related to various periodontal diseases. Human gingival fibroblast (HGF) is the major constituent in periodontal connective tissue and secretes various inflammatory mediators, such as nitric oxide (NO) and prostaglandin E_2_ (PGE_2_), upon lipopolysaccharide stimulation. This study is aimed at investigating the anti-inflammatory mechanism of chlorogenic acid (CGA) on *Porphyromonas gingivalis* LPS- (LPS-PG-) stimulated HGF-1 cells. The concentration of NO and PGE_2_, as well as their responsible enzymes, inducible NO synthase (iNOS), and cyclooxygenase-2 (COX-2), was analyzed by Griess reaction, ELISA, and western blot analysis. LPS-PG sharply elevated the production and protein expression of inflammatory mediators, which were significantly attenuated by CGA treatment in a dose-dependent manner. CGA treatment also suppressed activation of Toll-like receptor 4 (TLR4)/myeloid differentiation primary response gene 88 (MyD88) and nuclear factor- (NF-) *κ*B in LPS-PG-stimulated HGF-1 cells. Furthermore, LPS-PG-induced phosphorylation of extracellular regulated kinase (ERK) and Akt was abolished by CGA treatment, while c-Jun N-terminal kinase (JNK) and p38 did not have any effect. Consequently, these results suggest that CGA ameliorates LPS-PG-induced inflammatory responses by attenuating TLR4/MyD88-mediated NF-*κ*B, phosphoinositide-3-kinase (PI3K)/Akt, and MAPK signaling pathways in HGF-1 cells.

## 1. Introduction

Inflammation refers to the physiological response against numerous types of damage, such as heat, chemical injury, and infection by microorganisms [1]. Among numerous lines of inflammatory disorders, periodontal disease is one of the major public health problems in the world which is characterized by chronic inflammation of periodontium [2]. The periodontal disease indicates a set of infectious inflammation issued by periodontopathic bacteria that is possible to devastate the tooth-supporting tissues, which can be classified as a gingival disease and periodontitis [3, 4]. Gingival disease means an inflammatory condition in the gingival tissues caused by the accumulation of dental plaque. Periodontitis is a severe gum disease accompanied by plaque-induced inflammation which can give damage the periodontal ligament and alveolar bone [1]. Among various pathogens that contribute to the progress of periodontitis, *Porphyromonas gingivalis*, an anaerobic Gram-negative rod-shaped bacterium, is considered as one of the main causes in the progression of periodontal inflammation [5]. This oral bacterium attacks the host's immune system in a variety of bioactive materials, including cytoplasmic membranes, peptidoglycans, lipopolysaccharides (LPS), and fimbriae [6]. LPS from *P. gingivalis* (LPS-PG) has been regarded as a critical pathogenic component during the onset and development of periodontal disease, in which bacterial LPS can play a critical accelerator for the production of inflammatory cytokines and bone resorption [6, 7].

Human gingival fibroblast (HGF) is one of the main cell types located in periodontal tissue and can overproduce various inflammatory mediators, such as nitric oxide (NO), prostaglandin E_2_ (PGE_2_), and interleukins (ILs) when Toll-like receptor 4 (TLR4) is stimulated upon LPS-PG exposure [8]. The TLR4 is activated by LPS exposure and transduces its signal to myeloid differentiation primary response gene 88 (MyD88) to downstream signaling molecules for inflammation in HGF [9]. Thus, elevated inflammatory responses by LPS-PG can promote the severity of periodontal disease, and downregulation of LPS-initiated TLR4/MyD88-mediated inflammatory mediators could be a promising strategy for periodontitis [10].

Chlorogenic acid (CGA) is a well-known phenolic acid compound that is abundantly found in burdock, artichoke, eucommia, coffee beans, and tea [11]. It has been reported that CGA exerts various pharmacological activities such as anti-inflammatory, antioxidative, antibacterial, hepatoprotective, neuroprotective, and lipid modulatory effects [12]. In the field of dental pharmacology, CGA exhibited antimicrobial activity through the inhibited proliferation and protease activity of *P. gingivalis* [13]. Furthermore, CGA inhibited osteoclastic bone resorption by a downregulated receptor activator of nuclear factor- (NF-) *κ*B ligand- (RANKL-) induced osteoclast differentiation and LPS-induced bone loss [14]. Aqueous extract from the leaves of *Rhododendron ferrugineum*, containing 1.6% CGA, attenuated both the production of inflammatory cytokines induced by *P. gingivalis* in epithelial buccal KB cells and adhesion to KB cells [15]. Despite there being many trials to analyze the role of CGA in periodontitis, the exact anti-inflammatory mechanisms in HGF have not been understood yet. Therefore, the present study is aimed at investigating the anti-inflammatory mechanisms of CGA in LPS-PG-stimulated HGF-1 cells.

## 2. Materials and Methods

### 2.1. Reagents

Dulbecco's Modified Eagle Medium (DMEM) and fetal bovine serum (FBS) were purchased from Cytiva (Marlborough, MA, USA). LPS-PG was obtained from Invivogen (San Diego, CA, USA). MG-132, LY294002, U0126, and CGA were purchased from Sigma-Aldrich (St. Louis, MI, USA) which were dissolved in dimethyl sulfoxide (DMSO).

### 2.2. Cell Culture and Treatment

The HGF-1 cell line (CRL-2014, American Type Culture Collection, Manassas, VA, USA) was cultured in DMEM supplemented with 10% FBS, 2 mM L-glutamine, penicillin (100 U/mL), and streptomycin (100 *μ*g/mL).

### 2.3. Cell Viability Assay

Cell viability was determined by the CellTiter 96 Aqueous one solution cell proliferation assay (Promega Corporation, Madison, WI, USA). HGF-1 cells were seeded in a 24-well plate (5 × 10^4^ cells/well) and incubated with or without various concentrations of CGA for 24 h. Fifty microliters of MTS solution were added to 950 *μ*L of DMEM and incubated for 1 h at 37°C; then, the absorbance was measured at 490 nm with an xMark Microplate Absorbance Spectrophotometer (Bio-Rad Laboratories, Hercules, CA, USA).

### 2.4. Griess Reaction for NOS Activity Determination

HGF-1 cells were seeded in a 6-well plate (2 × 10^5^ cells/well) and preincubated with various concentrations of CGA for 2 h. Then, 1 *μ*g/mL of LPS-PG was added and incubated for 12 h, the optimal time for the induction of inflammation in HGF-1 cells (Supplementary Figure 1), for NOS induction. For NOS activity measurement in cell lysates, HGF-1 cells were lysed by three times of freeze-thaw cycle in 0.1 mL of 40 mM Tris buffer (pH 8.0) containing 5 *μ*g/mL of pepstatin A, 1 *μ*g/mL of chymostatin, 5 *μ*g/mL of aprotinin, and 100 *μ*M phenylmethylsulfonyl fluoride. The protein concentration was determined by the Bradford assay. NOS enzyme activity was measured as previously described [16]. Briefly, 20 *μ*g protein was incubated in 20 mM Tris–HCl (pH 7.9) containing 4 *μ*M FAD, 4 *μ*M tetrahydrobiopterin, 3 mM DTT, and 2 mM each of L-arginine and NADPH. The reaction was performed in triplicate for 3 h at 37°C on a 96-well plate. Residual NADPH was oxidized enzymatically, and the Griess reaction was performed.

### 2.5. PGE_2_ Concentration

The concentration of PGE_2_ in the supernatant was determined using an ELISA kit (Cayman Chemical, Ann Arbor, MI, USA) followed by the manufacturer's instructions.

### 2.6. Western Blot Analysis

Cells (2 × 10^6^ cells/dish) in 100 mm plates were preincubated with and without indicated concentrations of each sample for 2 h and then incubated with LPS-PG (1 *μ*g/mL) for 18 h. Cells were washed twice with PBS and scraped into 0.4 mL of protein extraction solution (M-PER, Thermo Fisher Scientific, Waltham, MA, USA) for 10 min at room temperature. The lysis buffer containing the disrupted cells was centrifuged at 13,000 × g for 10 min. Protein samples (25 *μ*g) from each lysate were separated on a 10% SDS polyacrylamide gel and electrotransferred to a PVDF membrane (Bio-Rad Laboratories). Primary antibodies were then incubated at 4°C overnight with a 1 : 1000 dilution after the membrane blocking for 1 h at room temperature with 5% nonfat dry milk in TBST solution. Then, the membrane was incubated with a 1 : 1000 dilution of HRP-conjugated anti-rabbit IgG (Cell Signaling Technology, Danvers, MA, USA) for 2 h at room temperature. The blots were developed with ECL developing solution (Santa Cruz Biotechnology, Santa Cruz, CA, USA), and data were quantified using the Gel Doc EQ System (Bio-Rad Laboratories). The primary antibodies were as follows: anti-inducible NO synthase (iNOS, 1 : 1000), anti-cyclooxygenase-2 (COX-2, 1 : 1000), anti-phospho-p65 (1 : 1000), anti-p65 (1 : 1000), anti-phospho-Akt (1 : 1000), anti-Akt (1 : 1000), anti-phospho-extracellular signal-regulated kinase (ERK, 1 : 1000), anti-ERK (1 : 1000), anti-phospho-cjun N-terminal kinase (JNK, 1 : 1000), anti-JNK (1 : 1000), anti-phospho-p38 (1 : 1000), anti-p38 (1 : 1000), anti-actin (1 : 1000), anti-TLR4 (1 : 1000), and anti-MyD88 (1 : 2000). All antibodies were obtained from Cell Signaling Technology and Abcam (Cambridge, UK).

### 2.7. Statistical Analysis

All data are expressed as means ± S.D. Statistical analyses were performed with SPSS version 25.0 (IBM Corp., Armonk, NY, USA). One-way ANOVA with Tukey's multiple comparison test was used to analyze the difference between each group. *p* < 0.05 was considered to indicate a statistically significant difference.

## 3. Results

### 3.1. Effect of CGA on the Production of Inflammatory Mediators in LPS-PG-Induced HGF-1 Cells

**Figure 1:**
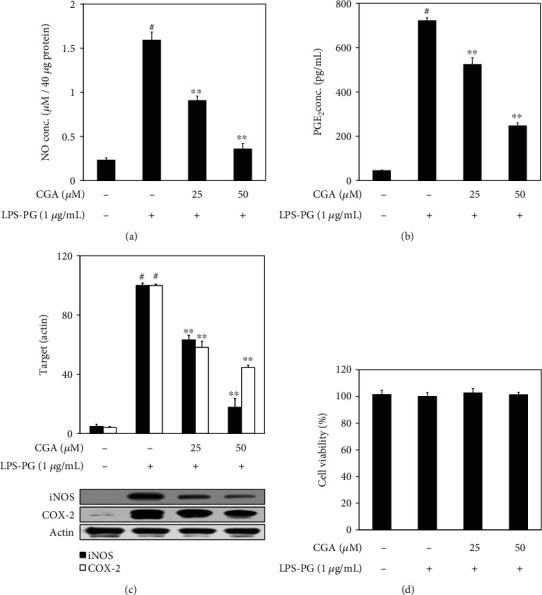
Attenuated production of NO and PGE_2_ as well as protein expression levels of their responsible enzymes, iNOS, and COX-2, by CGA treatment in LPS-PG-stimulated HGF-1 cells. Cells were preincubated with or without the indicated concentrations of CGA for 2 h, then incubated with LPS-PG (1 *μ*g/mL) for 18 h at 37°C in a humidified atmosphere containing 5% CO_2_. Griess reaction, ELISA, and western blot analysis were applied to measure the concentration of inflammatory mediators and protein expression levels. CGA treatment significantly inhibited LPS-PG-stimulated NO (a) and PGE_2_ production (b) as well as protein expression of iNOS and COX-2 (c) in HGF-1 cells. In addition, the cell viability was evaluated by MTS assay, and CGA treatment did not give any cytotoxic effect in HGF-1 cells (d). The relative protein expression of each target was measured by densitometry and normalized to the protein levels of actin, an internal control. Data represent the mean ± SD of triplicate experiments. ^#^*p* < 0.01 vs. NC group; ^∗^*p* < 0.05 and ^∗∗^*p* < 0.01 vs. LPS-PG group. Negative control (NC) group refers that CGA and LPS-PG were not treated. CGA: chlorogenic acid; COX-2: cyclooxygenase-2; HGF: human gingival fibroblast; iNOS: inducible nitric oxide synthase; LPS-PG: lipopolysaccharide from *P. gingivalis*; PGE_2_: prostaglandin E_2_.

The present study is aimed at investigating the anti-inflammatory mechanism of CGA in LPS-PG-stimulated HGF-1 cells. To demonstrate the anti-inflammatory activity of CGA in LPS-PG-stimulated HGF-1 cells, the Griess reaction and ELISA were applied to determine the concentration of NO and PGE_2_ in the supernatant. As shown in Figures 1(a) and 1(b), LPS-PG treatment potently induced acute inflammation, reflected by exaggerated NO and PGE_2_ production, was dose-dependently attenuated by CGA treatment without any cytotoxicity (Figure 1(d)) in HGF-1 cells. In addition, western blot analysis was applied to evaluate the protein expression levels of iNOS and COX-2, which was also significantly inhibited by CGA treatment in a dose-dependent manner (Figure 1(c)).

### 3.2. Effect of CGA on the Expression of TLR4/MyD88 and NF-*κ*B in LPS-PG-Induced HGF-1 Cells

**Figure 2:**
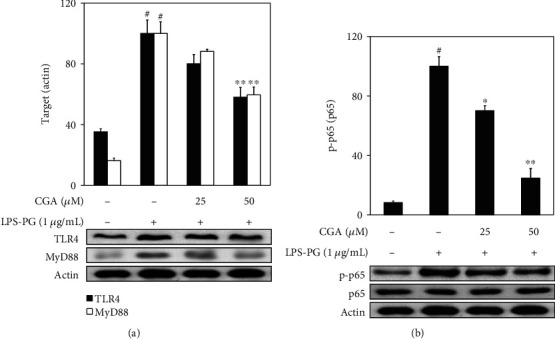
Inhibited TLR4/MyD88 and NF-*κ*B activations by CGA treatment in LPS-PG-stimulated HGF-1 cells. Cells were treated with the indicated concentrations CGA and LPS-PG (1 *μ*g/mL) for 4 h at 37°C in a humidified atmosphere containing 5% CO_2_ in order to analyze the TLR4/MyD88 and NF-*κ*B activations. CGA treatment significantly mitigated LPS-PG-induced TLR4/MyD88 and phosphorylation of p65, one subunit of NF-*κ*B, stimulation in HGF-1 cells. The relative protein expression of each target was measured by densitometry and normalized to protein levels of actin, an internal control. Data represent the mean ± SD of triplicate experiments. ^#^*p* < 0.01 vs. NC group; ^∗^*p* < 0.05 and ^∗∗^*p* < 0.01 vs. LPS-PG group. Negative control (NC) group refers that CGA and LPS-PG were not treated. CGA: chlorogenic acid; HGF: human gingival fibroblast; LPS-PG: lipopolysaccharide from *P. gingivalis*; MyD88: myeloid differentiation primary response gene 88; NF-*κ*B: nuclear factor-*κ*B; TLR4: Toll-like receptor 4.

TLR4 initially can recognize LPS and transduce the signal to MyD88 that is possible to activate NF-*κ*B signaling pathway [9]. NF-*κ*B plays a critical role in the production of inflammatory mediators in gingival fibroblasts [17]. To analyze the anti-inflammatory mechanism of CGA in LPS-PG-stimulated HGF-1 cells, TLR4/MyD88-mediated NF-*κ*B signaling pathway was estimated by western blot analysis. As shown in Figure 2, LPS-PG treatment significantly increased the expression of TLR4/MyD88 and p65 phosphorylation, which was dose-dependently attenuated by CGA treatment in HGF-1 cells.

### 3.3. Effect of CGA on LPS-PG Stimulated the Activation of PI3K/Akt and MAPK in HGF-1 Cells

**Figure 3:**
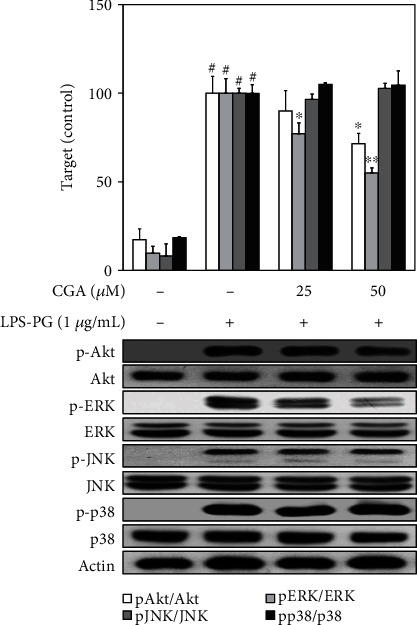
Attenuated phosphorylation of Akt and ERK by CGA treatment in LPS-PG-stimulated HGF-1 cells. Cells were treated with the indicated concentrations CGA and LPS-PG (1 *μ*g/mL) for 4 h at 37°C in a humidified atmosphere containing 5% CO_2_ in order to analyze the phosphorylation of signaling molecules. CGA treatment inhibited LPS-PG-stimulated phosphorylation of Akt and ERK in HGF-1 cells. The relative protein expression of each target was measured by densitometry and normalized to protein levels of unphosphorylated forms of each signaling molecule. Data represent the mean ± SD of triplicate experiments. ^#^*p* < 0.01 vs. NC group; ^∗^*p* < 0.05 and ^∗∗^*p* < 0.01 vs. LPS-PG group. Negative control (NC) group refers that CGA and LPS-PG were not treated. CGA: chlorogenic acid; JNK: c-Jun N-terminal kinase; ERK: extracellular regulated kinase; HGF: human gingival fibroblast; LPS-PG: lipopolysaccharide from *P. gingivalis*.

**Figure 4:**
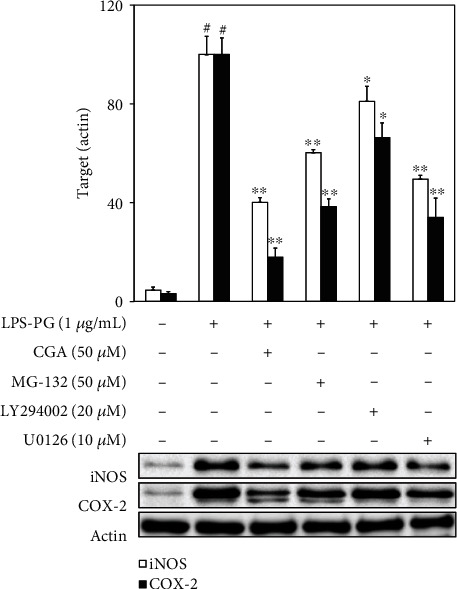
Inhibited iNOS and COX-2 expressions by selective inhibitor treatment in LPS-PG-stimulated HGF-1 cells. Cells were treated with the indicated concentrations LPS-PG, CGA, MG-132, LY294002, and U0126 for 18 h at 37°C in a humidified atmosphere containing 5% CO_2_ in order to confirm the anti-inflammatory mechanism of CGA in HGF-1 cells. The relative inhibition of iNOS and COX-2 was quantified by densitometry, and actin was used as an internal control. Data represent the mean ± SD of triplicate experiments. ^#^*p* < 0.01 vs. NC group; ^∗^*p* < 0.05 and ^∗∗^*p* < 0.01 vs. LPS-PG group. Negative control (NC) group refers that CGA, LPS-PG, and selective inhibitors were not treated. LPS-PG: lipopolysaccharide from *P. gingivalis*; CGA: chlorogenic acid; iNOS: inducible nitric oxide synthase; COX-2: cyclooxygenase-2; HGF: human gingival fibroblast.

Western blot analysis was used to analyze the phosphorylated status of phosphoinositide 3-kinase (PI3K)/Akt as upstream signaling molecules for NF-*κ*B and mitogen-activated protein kinase (MAPK) downstream of MyD88, which can regulate inflammatory responses in HGF-1 cells. This study tried to examine the inhibitory effect of CGA related to the PI3K/Akt, and MAPK signaling cascades were analyzed in LPS-PG-stimulated HGF-1 cells. As shown in Figure 3, CGA significantly inhibited phosphorylation of Akt and ERK in a dose-dependent manner, while other signaling molecules were not influenced by CGA treatment. Furthermore, the selective inhibitor of each signaling molecule was applied to analyze the role of NF-*κ*B, PI3K, and ERK molecules in inflammatory cascades stimulated by LPS-PG. Indicated concentrations of MG-132, LY294002, and U0126 and selective inhibitors of NF-*κ*B, PI3K, and ERK, respectively, significantly inhibited iNOS and COX-2 expression in LPS-PG-stimulated HGF-1 cells [18–20] (Figure 4). These results suggest that CGA significantly inhibited LPS-PG-induced inflammatory response through the regulation of TLR4/MyD88-mediated PI3K/Akt/NF-*κ*B activation and ERK phosphorylation in LPS-PG-stimulated HGF-1 cells.

## 4. Discussion

The inflammatory response in periodontal tissue is a complex defense mechanism that can be triggered by periodontopathic bacteria such as *Aggregatibacter actinomycetemcomitans*, *Prevotella intermedia*, and *P. gingivalis* [1]. Among them, *P. gingivalis*, a Gram-negative anaerobe, is one of the main pathogens that colonize dental plaque in the human oral cavity and acts as a major cause of chronic periodontitis [21]. Prolonged periodontitis can destroy the alveolar bone and its supporting tissues that lead to gum retrogression, bone weakness, and eventual tooth loss in adults [22]. The pathogenic properties of *P. gingivalis* are initiated from the various virulence factors such as lipopolysaccharide, fimbria, and gingipain [21]. LPS is a component of the outer membrane of Gram-negative bacteria and can stimulate HGF in periodontal tissue. Among various types of cells in the periodontium, HGF is a major cell type consisting of human gingival connective tissue and plays an important role in the development of periodontal inflammation through the exaggerated expression of iNOS and COX-2, the enzymes responsible for NO and PGE_2_, in response to exposure to LPS [2, 23, 24]. NO is produced by the deamination of L-arginine by NOS and consisted of 3 distinct isoforms including neuronal NOS (nNOS or NOS1), inducible NOS (iNOS or NOS2), and endothelial NOS (eNOS or NOS3). Among them, iNOS is produced by various inflammatory stimuli, such as bacterial LPS exposure, TNF-*α*, IL-6, and IL-8 release, while eNOS and nNOS maintain normal physiological reactions [25]. An appropriate amount of NO in periodontal tissue may play a role for the nonspecific natural defense mechanisms in the oral cavity but excessively generated NO could destroy local tissue in periodontitis lesions and exacerbate pathogenesis of the periodontal inflammatory disease [26]. Cyclooxygenase catalyzes the conversion of arachidonic acid to prostaglandins and is composed of two distinct enzymes, COX-1 and COX-2. COX-1 plays a role in maintaining cellular homeostasis while COX-2 is potently induced by inflammatory and other physiological stimuli [27]. Especially, exaggerated PGE_2_ production and its responsible enzyme, COX-2, overexpression in periodontal tissue were recognized as critical hallmarks of exacerbated periodontal inflammation [28, 29]. This study employed LPS-PG to induce inflammatory responses in HGF-1 cells, which was reflected by the accelerated NO and PGE_2_ productions as well as the increased expression of their corresponding enzymes, iNOS, and COX-2. By the way, CGA treatment dose-dependently attenuated exaggerated production and protein expression of both inflammatory mediators in LPS-PG-stimulated HGF-1 cells as shown in Figure 1. This means that CGA has the activity to attenuate LPS-PG-induced inflammatory mediators, which have the potential to progress periodontitis, in HGF-1 cells.

The immune defense system against pathogens is initiated from their perception by highly conserved PRRs, including TLRs [30]. TLRs are a growing family that activates innate immunity and inflammatory responses upon the interaction with numerous pathogen-associated molecular patterns including bacterial LPS, viral RNA, and flagellin [31]. HGF expresses TLR2, 4, and 5 for a critical role in immune response, principally faces and interacts with pathogenic invasion at an early stage of periodontitis [27, 32]. As a ligand for TLR4, LPS can bind to the extracellular domain of TLR4 and form intracellular adaptor molecules, including the adaptor protein containing MyD88 and Toll-interleukin 1 receptor domain (TIRAP) (31, 33, 34). Accelerated production of MyD88 can lead to the activation of NF-*κ*B, PI3K/Akt, and MAPKs and the production of inflammatory mediators [33–35]. NF-*κ*B, the inflammatory transcription factor, is involved in the regulation of inflammation, cell proliferation, the immune system, and differentiation. This transcription factor exists ubiquitously in the cytoplasm in an inactive form and can be induced by bacterial infection, inflammatory cytokines, UV irradiation, and oxidative stress [36]. NF-*κ*B consists of p65 and p50 subunits that are anchored by the inhibitor protein, I*κ*B*α* [37]. In response to stimuli, this transcription factor can be converted into the activated form through the phosphorylation of the NF-*κ*B subunit, p65. The activated form of p65, phospho-p65, can translocate to the nucleus and bind to the promoter region for transcription of various inflammation-related genes [37, 38]. As the upstream signaling molecule of NF-*κ*B, PI3K/Akt and MAPK signaling pathways are critical regulators of the production of LPS-induced inflammatory mediators that can play a role in the progression of periodontitis [17]. This study attempted to investigate the anti-inflammatory mechanisms of CGA in human periodontitis. The activated status of TLR4/MyD88, PI3K/Akt, and MAPKs was analyzed to make clear the regulation of upstream signaling molecules related to NF-*κ*B modulation in LPS-PG-stimulated HGF-1 cells. CGA treatment attenuated LPS-PG-induced TLR4/MyD88 expression in a dose-dependent manner, which means that the anti-inflammatory effect of CGA in HGF-1 cells is associated with the TLR4/MyD88 signaling pathway (Figure 2(a)). Phosphorylated p65, a subunit of NF-*κ*B, was also attenuated by CGA treatment, which was in accordance with the result of TLR4/MyD88 expression (Figure 2(b)). The phosphorylated status of Akt, ERK, JNK, and p38 was estimated by western blot analysis and shown in Figure 3. Treatment with CGA inhibited ERK phosphorylation but did not give any effect on PI3K/Akt, JNK, and p38 activations. Furthermore, specific inhibitors against NF-*κ*B, PI3K, and ERK were applied to confirm the inhibitory mechanism of CGA in LPS-PG-induced inflammatory responses in HGF-1 cells (Figure 4). These results indicate that CGA significantly ameliorates LPS-PG-stimulated inflammatory mediators through the regulation of TLR4/MyD88-mediated PI3K/Akt/NF-*κ*B and MAPK signaling pathways in HGF-1 cells. In a further study, the investigation of the exact anti-inflammatory mechanisms of CGA has to be evaluated in periodontitis animal models.

## Data Availability

Data used to support the findings of this study are available from the corresponding author upon request.

## Supplementary Materials

Supplementary Materials Supplementary Figure 1: the protein expression level of iNOS by time course in LPS-PG-stimulated HGF-1 cells.
