# Supplementary material for: Chlorogenic Acid as a Positive Regulator in LPS-PG-Induced Inflammation via TLR4/MyD88-Mediated NF-κB and PI3K/MAPK Signaling Cascades in Human Gingival Fibroblasts
Source: Mediators Inflamm. 2022 Apr 9;2022:2127642. doi: 10.1155/2022/2127642 (PMC9013303; doi:10.1155/2022/2127642)
Supplement: Supplementary Materials — Supplementary Figure 1: the protein expression level of iNOS by time course in LPS-PG-stimulated HGF-1 cells. [file 2127642.f1.docx]

|  | | | | | |
| --- | --- | --- | --- | --- | --- |
| Treatment time (h) | 0 | 6 | 12 | 24 | 48 |
| LPS-PG (1 μg/mL) | - | + | + | + | + |
| iNOS | 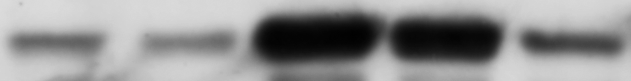 | | | | |
| Actin | 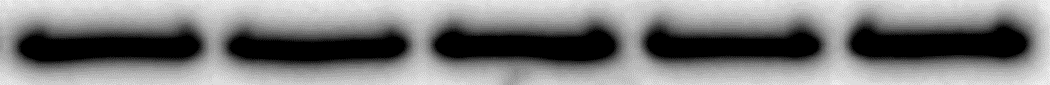 | | | | |

Supplementary Figure 1: The protein expression level of iNOS by time course in LPS-PG-stimulated HGF-1 cells. Cells were incubated with the indicated concentration of LPS-PG (1 *μ*g/mL) at 37˚C in a humidified atmosphere containing 5% CO_2_. Western blot analysis was applied to measure the expression level of iNOS. The relative protein expression was measured by densitometry and normalized to the protein levels of actin, an internal control. Data represent the mean±SD of triplicate experiments. ^*^p<0.05 and ^**^p<0.01 *vs*. NC group. Negative control (NC) group refers that LPS-PG was not treated. HGF, human gingival fibroblast; iNOS, inducible nitric oxide synthase; LPS-PG, lipopolysaccharide from *P. gingivalis*.
